# Epistemology for interdisciplinary research – shifting philosophical paradigms of science

**DOI:** 10.1007/s13194-018-0242-4

**Published:** 2018-12-12

**Authors:** Mieke Boon, Sophie Van Baalen

**Affiliations:** Department of Philosophy, University of Twente, Enschede, The Netherlands

**Keywords:** Interdisciplinarity, Problem-solving, Epistemological views, Disciplinary matrix, Kuhn, Disciplinary perspectives, Engineering paradigm of science, Engineering sciences, Higher education, Expertise, Metacognitive skills, Higher-order cognitive skills, Metacognitive scaffolds

## Abstract

In science policy, it is generally acknowledged that science-based problem-solving requires interdisciplinary research. For example, policy makers invest in funding programs such as Horizon 2020 that aim to stimulate interdisciplinary research. Yet the epistemological processes that lead to effective interdisciplinary research are poorly understood. This article aims at an epistemology for interdisciplinary research (IDR), in particular, IDR for solving ‘real-world’ problems. Focus is on the question why researchers experience cognitive and epistemic difficulties in conducting IDR. Based on a study of educational literature it is concluded that higher-education is missing clear ideas on the epistemology of IDR, and as a consequence, on how to teach it. It is conjectured that the lack of philosophical interest in the epistemology of IDR is due to a *philosophical* paradigm of science (called a *physics paradigm of science*), which prevents recognizing severe epistemological challenges of IDR, both in the philosophy of science as well as in science education and research. The proposed alternative philosophical paradigm (called an *engineering paradigm of science*) entails alternative philosophical presuppositions regarding aspects such as the aim of science, the character of knowledge, the epistemic and pragmatic criteria for accepting knowledge, and the role of technological instruments. This alternative philosophical paradigm assume the production of knowledge for epistemic functions as the aim of science, and interprets ‘knowledge’ (such as theories, models, laws, and concepts) as *epistemic tools* that must allow for conducting epistemic tasks by epistemic agents, rather than interpreting knowledge as *representations* that objectively represent aspects of the world independent of the way in which it was constructed. The engineering paradigm of science involves that knowledge is indelibly shaped by how it is constructed. Additionally, the way in which scientific *disciplines* (or fields) construct knowledge is guided by the specificities of the discipline, which can be analyzed in terms of *disciplinary perspectives*. This implies that knowledge and the *epistemic uses* of knowledge cannot be understood without at least some understanding of how the knowledge is constructed. Accordingly, scientific researchers need so-called *metacognitive scaffolds* to assist in analyzing and reconstructing how ‘knowledge’ is constructed and how different disciplines do this differently. In an engineering paradigm of science, these metacognitive scaffolds can also be interpreted as epistemic tools, but in this case as tools that guide, enable and constrain analyzing and articulating *how* knowledge is produced (i.e., explaining epistemological aspects of doing research). In interdisciplinary research, metacognitive scaffolds assist interdisciplinary communication aiming to analyze and articulate how the discipline constructs knowledge.

## Introduction

### Background

Most current scientific research in the natural, medical and engineering sciences is expected to be useful for specific ‘real-world’ problems, such as in agriculture and food-industry, (bio)medical technology and pharmacy, ICT and transport, civil engineering and sustainability, weather-forecasting and climate issues, safety and forensics, energy-technology and high-tech (nano-)materials. Real-world problems usually are complex, and philosophers of science emphasize that this requires interdisciplinary or transdisciplinary research (e.g., Schmidt 2008, 2011; Frodeman 2010; Krohn 2010; Alvargonzález 2011; Thorén and Persson 2013; Thorén 2015; Maki 2016; O'Rourke et al. 2016). Outside the philosophy of science, the need for interdisciplinary research for real-world problem solving —and associated changes in the structure of universities and higher education systems— has been stressed for decades already (e.g., Jantsch 1972; Apostel et al. 1972; Klein 1990, 1996; Kline 1995; Aram 2004; National Academy of Sciences 2005; Hirsch-Hadorn et al. 2010; Bammer 2013; and see overviews by Jacobs and Frickel 2009, and Newell 2001, 2013). These authors, specialized in science policy and education studies, also report that attempts to promote interdisciplinary research at universities and to train university students for conducting interdisciplinary research often do not lead to the desired results. Scholars with a policy or sociology background usually explain difficulties in establishing interdisciplinary research and education in terms of organization and tend to deny epistemological and cognitive factors.

In the philosophy of science, interdisciplinary research is studied, but with a few exceptions, hardly any philosophical research has been conducted into ‘why interdisciplinary research is so difficult’ (MacLeod 2016; Thorén 2015). The same applies to educational research, in which both conceptual studies and empirical research are conducted into higher education programs that train students in interdisciplinary (or transdisciplinary) approaches – in these studies too little attention is paid to the cognitive and epistemological difficulties encountered by researchers, in particular, regarding the question whether the ability to *do* interdisciplinary research requires a specific understanding of science and related cognitive skills.

The thesis put forward in this article consists of two parts. The first part is that the neglect of cognitive and epistemological difficulties in *doing* interdisciplinary research is, at least partly, due to philosophical beliefs about science that still guide the higher education of researchers and professionals. Addressing this claim requires to explain why interdisciplinary research is difficult, and to show that epistemological and cognitive difficulties have to do with philosophical presuppositions about science, regarding traditional themes such as reductionism, the unity of science, epistemology as ‘evidence for justified true belief,’ and the distinction between the context of discovery and the context of justification. We will argue that, in order to deal with the epistemological and cognitive difficulties, alternatives to these presuppositions must be sought that are better suit to understanding interdisciplinary research.

The second part of our thesis is that an alternative philosophical view of science can be based on Kuhn’s idea of disciplinary matrices (Kuhn 1970), in particular, on an expanded version of elements constituting the matrix. This expanded matrix has been used to articulate two different philosophical visions of science, called a *physics paradigm of science* versus an *engineering paradigm of science* (Boon 2017a). Articulating these paradigms was intended to interpret the changing character of the biomedical sciences such as *systems biology* as compared to, for instance, classical biochemistry – and it was found that the engineering paradigm of science suits better to systems biology than the traditional physics paradigm. Extending this approach, we will argue that the *engineering paradigm of science* also fits better in understanding interdisciplinary scientific research in ‘real-world’ problem-solving contexts. The engineering paradigm of science makes it also possible to reconsider presuppositions associated with education in interdisciplinary research, which will be substantiated and illustrated by proposing enriched interpretations of some relevant concepts, such as ‘disciplinary perspectives,’ ‘higher-order cognitive skills’ (also called ‘metacognitive skills’), and ‘conceptual frameworks’ (in particular so-called ‘metacognitive scaffolds’).

Altogether, the aim of this article is to develop a vocabulary (a philosophical and conceptual framework) that enables detailed philosophical study of interdisciplinary research practices —particularly in ‘real-world’ problem-solving context— that takes into account the epistemic tasks and cognitive challenges as an inherent aspect of these practices, and also to indicate directions of how philosophers of science may contribute to the academic education therein. In developing this vocabulary, use is made of literature in scholarly domains ranging from philosophy of science, philosophy of education, science policy studies, and studies in (higher) education of science and engineering.

### Structure

The structure of this article is as follows. Section Two focuses on scholarly disciplines that study interdisciplinary research (IDR). These studies appear to focus on organizational aspects of IDR and have little interest in epistemological and cognitive difficulties of interdisciplinary research as experienced by individual researchers (MacLeod 2016). This lack of interest is obviously due to the specialization of these scholars (e.g. in science policy) but also stems from the conviction that suitable organization of IDR can solve problems of IDR – a belief that we dispute. In particular, most scholars conceive of IDR as aiming at the *integration* of knowledge, which we consider to be the epistemologically most challenging part of IDR. We will clarify this in terms of three metaphors of IDR.

Section Three deals with the kind of expertise needed in IDR, and presents educational insights and empirical findings about training scientific researchers and professionals in IDR. It focuses on views about the role of metacognitive skills. It appears that little is being done to train metacognitive skills for IDR, which is explained in terms of dominant philosophical views of science that guide science education, and which critical educational researchers call a *positivist* view of science.

Section Four first discusses the claim that traditional philosophical views of science insufficiently account for the *uses* of science, i.e., how scientific research *generates* and *applies* scientific knowledge (or, more generally phrased, *epistemic resources*) for solving real-world problems. This is illustrated by the fact that traditional philosophy of science has been much more interested in the *unity of science* while considering interdisciplinary research as a means for achieving unity but not as an issue relevant to philosophical study.

Subsequently, we will turn to the second part of our thesis, starting from the idea that the philosophy of science maintains philosophical views of science —which we call *philosophical* paradigms of science— that determine what subjects are worthy philosophical study and how these are studied. We argue that a *physics paradigm* of science has been dominant in traditional philosophy of science, which may have strengthened a physics-dominated view of science in science education and scientific research, and which currently impedes interdisciplinary research because it entails an ineffective epistemology that also conceals cognitive and epistemological difficulties. A more appropriate alternative epistemology comes from an *engineering paradigm of science*. An outline will be given of the extended Kuhnian matrix that is used as a conceptual framework to analyze philosophical views of science, and of the two philosophical paradigms that result from *using* this matrix as a framework to articulate philosophical views of science (Boon 2017a). Central is the difference between science considered as a unified hierarchy, or network of theories (in a physics paradigm), versus ‘knowledge’ considered as epistemic tools constructed and shaped within scientific disciplines, where epistemic tools must be constructed such as to be suitable for being used as epistemic resources in performing epistemic tasks in which new epistemic results are generated in an ever ongoing scientific research processes (in an engineering paradigm). Next, it is argued that the way in which ‘knowledge’ is constructed is partly determined by the *disciplinary perspective* within which experts perform their research. Kuhn’s traditional notion of *disciplinary matrices* is proposed as a framework to analyze disciplinary perspectives of disciplines.

Accepting the idea put forward in the engineering paradigm that ‘epistemic results’ such as scientific models are shaped by the specific disciplinary perspective of discipline D_A_ and therefore indelibly entail specificities of the discipline —instead of scientific models being more or less objective or ‘literal’ representations of aspects of the world, as is the epistemological ideal of a physics paradigm— explains why epistemic results produced in D_A_ usually do not speak for themselves and cannot be understood or used in a straightforward manner by experts in D_B_. It is concluded that: (a) the epistemology that results from the engineering paradigm points to fundamental reasons for the epistemological and cognitive difficulties that we aim to explain, and (b) that this explanation is not easily recognizable nor appreciated in a physics paradigm of science.

Finally, in Section 5, expanding on the engineering paradigm of science in which epistemic recourses (such as theories, models, laws, and concepts) are interpreted as *epistemic tools* for performing epistemic tasks, we will propose to also interpret the *metacognitive scaffolds* (discussed in this article) as epistemic tools. Similar to how ‘knowledge’ interpreted as epistemic tool allows, guides and constrains, for instance, thinking about aspects of the physical world, metacognitive scaffolds interpreted as epistemic tools allow, guide and constrain thinking about (epistemological) aspects of scientific practice, such as how the disciplinary perspective of D_A_ shapes the production of ‘knowledge’ in D_A_. This idea is rather programmatic but aims to make plausible that the epistemology emerging from the engineering paradigm of science not only explains epistemological and cognitive difficulties of interdisciplinary research but also indicates directions to mitigate these difficulties. This epistemology, therefore, is also very promising for developing metacognitive scaffolds that assist in learning and doing scientific research.

### Terminology

Throughout this article, we will use the terms ‘knowledge,’ ‘epistemic resources’ and ‘epistemic results’ interchangeably. In the text, ‘knowledge’ is often put between quotes to indicate that it encompasses all possible types of scientific knowledge, such as fundamental principles; theoretical and empirical knowledge about phenomena; scientific models, laws, concepts, and ‘descriptions’ of (unobservable) phenomena; practical knowledge about (observable) phenomena (e.g., on how to generate or causally affect them by physical or technological circumstances, see Boon 2017c); theoretical knowledge of the inner working of technological instruments and measurement apparatus (often represented in models of the instrument); and, knowledge of methodologies and epistemic strategies in scientific research.

In this article, we aim to add ‘metacognitive knowledge’ (i.e., the last item in the list above), which is knowledge not about the ‘real-world’ but about how to learn and how to do scientific research. This entails knowledge of epistemological views (e.g., as taught in philosophy of science courses) as well as knowledge of methodologies and epistemic strategies. This kind of knowledge is represented by means of so-called metacognitive scaffolds (also called matrices, or, frameworks) that can be utilized in learning and executing (interdisciplinary) research.

Finally, ‘epistemic resources’ is the ‘knowledge’ used in generating new ‘knowledge,’ while ‘epistemic results’ is the generated ‘knowledge.’ Epistemic results are also referred to as ‘epistemic entities.’ The first two terms emphasize that in this article, scientific research is considered as a never ending process of producing and using ‘knowledge,’ while ‘epistemic entity’ stresses that knowledge is used in a *tool-like fashion*. The engineering paradigm of science proposed in this article expresses a view of scientific research in which scientific researchers produce ‘knowledge’ that can be used in performing epistemic tasks for specific epistemic purposes (within or outside science), instead of science being the quest for complete and true knowledge.

## Studies of interdisciplinary research

### Definitions of inter- and transdisciplinary research

Interdisciplinary research is promoted, performed, administered, organized and taught, to the effect that these aspects are studied in scholarly domains ranging from science policy studies, governance studies, STS (science, technology and society) studies, science education, cognitive sciences, philosophy of science and social epistemology.

Already in the 1970s, Jantsch stressed the role of universities in achieving social goals and therefore called for a reform of universities and university education. Changes in society require that the university develops increasingly interdisciplinary approaches, which must also be reflected in university education (Jantsch 1972). Jantsch’s scholarly focus was the *organizational* structure of universities, and how by reforming this structure, interdisciplinarity for meeting societal goals can be achieved.

One of the aims of studies in interdisciplinarity is a correct *definition* of interdisciplinary research (Newell 2013). A consensus definition drawn up in the 1990s is: “A process of answering a question, solving a problem, or addressing a topic that is too broad or *complex* to be dealt with adequately by a single discipline or profession . . . [interdisciplinary research] draws on *disciplinary perspectives* and *integrates* their insights into a more *comprehensive perspective*” (Newell 2013, 24, our emphasis). Definitions can differ with respect to aspects such as problem-definition, level of integration, and the elements integrated, for example, not only laws and theories, but also concepts, theoretical frameworks, methodologies, procedures, instruments, and data (e.g., Apostel et al. 1972; Klein 1996; Lattuca 2001; Aboelela et al. 2007; Huutoniemi et al. 2010). Most authors agree that the *complexity* of a problem (either a problem within science or a ‘real world’ problem) is why interdisciplinary research is necessary (Newell 2013).^1^

Several authors take *transdisciplinarity* as a higher form of interdisciplinarity in the sense of ‘being about more complex problems’ —such as problems for which technological solutions are developed that also need to take into account societal, economical, ethical and other humanities aspects— which usually requires to go beyond the confines of academic disciplines (e.g. Klein 2010; Hirsch-Hadorn et al. 2010; Schmidt 2008, 2011; Alvargonzález 2011; Bergmann 2012; Aneas 2015). Aneas (2015, 1719), for instance, states that: “Transdisciplinarity is a higher stage of disciplinary interaction. It involves a comprehensive framework that organizes knowledge in a new way and is based on cooperation among various sectors of society and multiple stakeholders to address complex issues around a new discourse.” Yet, in the scholarly literature, many examples that would comply with this definition are still called interdisciplinary,^2^ especially in the engineering education literature (e.g., National Academy of Science 2005; Nikitina 2006; Gnaur et al. 2015; Van den Beemt et al. under review) and in philosophical literature (e.g., Lattuca 2001; Frodeman and Mitcham 2007; Cullingan and Pena-Mora 2010; Tuana 2013; Lattuca et al. 2017).^3^

In sum, many studies focus on organizational and institutional obstacles to interdisciplinary research, rather than the cognitive and epistemological obstacles (e.g., Turner 2000; Jacobs and Frickel 2009; Turner et al. 2015; Newell 2013). Yet, central to most *definitions* of interdisciplinary and transdisciplinary research —intended to give direction to their *organization*— is the *integration* of *knowledge* (or more broadly, *epistemic resources*). Such integration is probably the hardest part for researchers and requires specific abilities or *expertise*.

### Methods for organizing interdisciplinary research

Several authors have proposed *methods* for the (internal) *organization* of interdisciplinary research by specifying steps in the research process. Klein (1990), Repko (2008) and Repko and Szostak (2017), and Menken and Keestra (2016), for instance, wrote book-length treatments of the interdisciplinary research process.

There appears much overlap between the three methods, especially between Klein (1990) and Repko (2008) / Repko and Szostak (2017), but there is also a remarkable difference as to the aim of interdisciplinary research processes. While Klein, Repko and Szostak focus on developing understanding about complex problems *outside* science, Menken & Keestra’s approach is oriented at problems *within* science.^4^ The method by Menken and Keestra (2016) is faithful to how ‘science-oriented’ scientific research practices are traditionally understood, by including the phases of ‘formulating research (sub-)*questions* and *hypotheses*,’ ‘setting-up the research methods and design’ and ‘performing the data collection and analysis’ within the interdisciplinary setting. In fact, their schema is an expansion of the well-known hypothetical-deductive method.^5^ In explaining interdisciplinary research, their three additions as compared to the HD-method are: the decisions that need to be taken on relevant disciplines; the establishment or choice of a comprehensive theoretical framework within which the participating disciplines need to be embedded; and, the *integration* of results and insight. Menken and Keestra’s (2016) schema is traditional in the sense that it focuses on testing hypothesis, with interdisciplinary theoretical insights ‘within science’ as the result; whereas the schemas presented by Klein (1990), and by Repko (2008) and Repko and Szostak (2017) focuses on outcomes that are relevant to solutions for the ‘real-world’ problems at which the research project aims.

### Metaphors of integration: Jigsaw-puzzle, conflict-resolution, and engineering-design

The methods to coordinate research processes discussed above adequately reflect the proper organization of processes of interdisciplinary research as commonly adopted in current research projects.^6^ However, our worry remains how *linking* and *integration* of ‘knowledge’ (i.e., *epistemic resources*) is understood in these methods.

Regarding the linking or integration of knowledge three kinds of metaphors can be distinguished: (a) the *jigsaw-puzzle metaphor* according to which integration means that pieces of ‘knowledge’ are fitted together without changing them; (b) the *conflict-resolution metaphor*, which focuses on apparent disagreements supposedly due to hidden presuppositions and confusion about basic concepts, as in, say, political discourse, and *communication* to resolve these disagreements; and (c) the *engineering-design* metaphor, which focuses on the *construction* of epistemic resources for specific epistemic tasks, usually requiring creative designer-like inventions to *combine* relevant but heterogeneous bits and pieces into a coherent ‘epistemic entity’ (e.g., a scientific model) within specific epistemic and pragmatic requirements related to the epistemic uses of the epistemic entity. Our idea is that studies of interdisciplinarity may be implicitly guided by epistemological views —loosely expressed by the jigsaw puzzle and the conflict-resolution metaphors—, which hinder an understanding of the epistemic and cognitive difficulties of interdisciplinary research oriented at solving real-world problems, for which the *engineering-design* metaphor aims to be an alternative.

Menken and Keestra’s (2016) method complies with a *jigsaw-puzzle metaphor* of integration, whereas the methods proposed by Klein (1990), Repko (2008) and Repko and Szostak (2017) are closer to a conflict-resolution metaphor, which interprets difficulties of integration as conflicts due to misunderstandings that can be resolved by *communication* and *reflection* in order to establish ‘common ground.’

Also philosophers who aim to facilitate difficulties in interdisciplinary research lean towards the *conflict-resolution metaphor* (e.g., Nikitina 2006; Strang 2009; Fortuin and van Koppen 2016; O'Rourke et al. 2016). Undeniably, these philosophers have achieved positive results by means of conceptual frameworks and tools to generate philosophical dialogue by which cross-disciplinary communication is improved, for instance to clarify concepts and background beliefs – and in this manner philosophers can make contributions to students’ and researchers’ ability to reflect on presuppositions.

In short, the methods for interdisciplinary research proposed in interdisciplinary studies do not sufficiently address the inherent epistemic and cognitive difficulties of integration. The philosophical approaches that aim to help researchers solve conceptual confusion are on the right track, but they do not yet touch the deeper epistemological issues. The detailed cases of interdisciplinary research in engineering and bioengineering practices investigated by Mattila (2005), Nersessian (2009), Nersessian and Patton (2009), and MacLeod and Nersessian (2013) can be taken as examples that suit the *engineering-design* metaphor – these are illustrative examples of the complexity of interdisciplinary research, but they do not yet offer any tools for learning how to do such research.

## Studies of teaching and learning interdisciplinary research

### Higher-order cognitive skills for expertise in interdisciplinary research

Definitions of interdisciplinary research discussed in Section 2 focus on the *knowledge* part, i.e., the integration of knowledge and other epistemic elements, but interdisciplinary research has also been defined in terms of types of *expertise* of researchers (e.g., Goddiksen 2014; Goddiksen and Andersen 2014) and types of interdisciplinary *collaborations* (e.g., Rossini and Porter 1979; Andersen and Wagenknecht 2013; Andersen 2016). This section will focus on the *skills* needed as a crucial part of the expertise to conduct interdisciplinary research (Goddiksen and Andersen 2014; Collins and Evans 2002, 2007; Goddiksen 2014).

Scholarly work that aims to articulate the specific skills needed for performing interdisciplinary research and how these skills can be trained and assessed, is mostly found in educational literature. Above, we claimed that, from an epistemological perspective *linking* and *integrating* is perhaps one of the severest challenges of utilizing highly-fragmented scientific disciplines in solving highly-complex ‘real-world’ problems. In such interdisciplinary research, having *expertise* means that scientific researchers are able to use, link and integrate ‘knowledge’ (i.e., epistemic resources including data, concepts, models, and theories) and methods from disciplines D_A_, D_B_ etc. such as to generate ‘knowledge’ for solving the ‘real-world’ problem.

One may expect that science and engineering education at an academic level have clear ideas on how linking and integration in interdisciplinary research is done, and how to teach it. However, although many studies on engineering education aim at teaching interdisciplinary problem-solving, a thorough analysis of the epistemological difficulties seems to be lacking. In part, in engineering education literature this lack is due to the specific interpretation of interdisciplinary problem-solving, less oriented at *integration of knowledge*, but rather on taking into account *societal values* and constraints – which, as suggested above, should rather be called transdisciplinary.

Dealing with broader societal issues usually imply skills that are considered ‘soft’, social, (inter)personal and professional skills, such as integrity, communication, courtesy, responsibility, positive attitude, professionalism, flexibility, teamwork, and work ethic (e.g., Robles 2012; National Science Foundation 2008; Haynes and Brown-Leonard 2010; Gnaur et al. 2015; Bosque-Perez et al. 2016; Chan et al. 2017; Lattuca et al. 2017), for which authors typically promote problem-based or case-based pedagogies and co-curricular activities. If *cognitive* or *epistemological* challenges of interdisciplinary research projects are mentioned at all, authors usually assume that this is solved by being trained in teamwork and communication skills (see Stentoft 2017 for a comprehensive review).

However, based on a critical meta-study (i.e., a systematic search within scientific literature databases) reviewing how ‘interdisciplinary thinking’ is taught in higher education more generally, Spelt et al. (2009) conclude that research on this topic is still limited. *Interdisciplinary thinking* according to them is “the capacity to integrate knowledge of two or more disciplines to produce a cognitive advancement in ways that would have been impossible or unlikely through single disciplinary means,” which they consider a *complex cognitive skill*. Other authors add that interdisciplinary thinking is a higher-order or metacognitive skill, which involves the ability to search, identify, understand, critically appraise, connect, and integrate theories and methods of different disciplines and to apply the resulting cognitive advancement together with continuous evaluation (e.g., Ivanitskaya et al. 2002; Lourdel et al. 2007; DeZure 2010; Zohar and Barzilai 2013; Goddiksen and Andersen 2014). Additionally, interdisciplinary thinking requires specific types of knowledge – i.e., next to having knowledge of one’s discipline, also knowledge of *disciplinary paradigms* and of interdisciplinarity is needed (Spelt et al. 2009).

Similarly, based on a meta-study as well, Khosa and Volet (2013) conclude that the intended *higher-order cognitive skills* needed in interdisciplinary research are not actually achieved through student-led collaborative and case-based learning activities at university level, and argue that students may need instruction to the development of these skills. In most education reported in the literature, teaching higher-order metacognitive skills (also referred to as ‘deep-learning’) is approached by inviting *self-reflection* of students. However, usually no clear guidance for how to do this is given. In a recent systematic review of engineering education literature on teaching interdisciplinarity, Van den Beemt et al. (under review) found that problem- and project-oriented (PBL) forms of education to promote interdisciplinarity, do indeed promote *societal and professional skills and attitudes*, including teamwork, project-management and communication, but these authors conclude that there are no indications that problem-based learning (PBL) approaches are successful with regard to the development of higher-order metacognitive skills needed for *linking* and *integrating* epistemic resources in real-world problem-solving. Educational programs in engineering often assume that these skills will be learned ‘by doing’ and do not need additional support. Similar to Khosa and Volet (2013), this assumption is contested by Zohar and Barzilai (2013), who argue that learning higher-order metacognitive skills needs to be supported by *metacognitive scaffolds*, which, according to these authors, are generally underdeveloped – i.e., hardly any such scaffolds have been developed. In Section 5, we will make some suggestions about possible scaffolds (or frameworks) to support the development of higher-order skills of students in conducting interdisciplinary research.

Summing up, in order to actually use and generate ‘knowledge’ for solving complex real-world problems, researchers need higher-order cognitive skills, because rules or algorithms for how to use a theory, model, concept or data in this respect usually are not given by the epistemic resources.^7^ Based on studies in the educational literature it is concluded that the teaching of higher-order cognitive skills as a crucial part of expertise in conducting interdisciplinary research remains underdeveloped in higher education, which may be an important reason for difficulties that researcher have in dealing with interdisciplinary research (Thorén and Persson 2013; Thorén 2015; MacLeod 2016). It now needs to be explained: (a) in what sense the lack of teaching these skills has to do with traditional epistemological views, and (b) how alternative epistemological views provide understanding of these specific higher-order cognitive skills.

### Epistemological views conveyed in science teaching

Before turning to the idea that traditional epistemological views may hamper educational ideas on how to teach higher-order cognitive skills as part of developing expertise in interdisciplinary research, the term ‘metacognitive skill’ (and the term ‘higher-order cognitive skill,’ which in this context is used interchangeably) requires some clarification. Flavell (1979) is a developmental psychologist who is said to have introduced the notion *metacognitive knowledge*, defined as one’s stored knowledge or beliefs about oneself and others as cognitive agents, about tasks, about actions or strategies, and about how all these interact to affect the outcomes. Metacognitive knowledge consists primarily of knowledge or beliefs about what factors or variables act and interact in what ways to affect the course and outcome of cognitive enterprises. There are three major categories of these factors or variables—person, task, and strategy (Flavell 1979, 907; also see Pintrich 2002). Hence, the original focus in the cognitive sciences was on knowledge concerning one’s own cognitive processes, called *metacognitive knowledge*. Additionally, it involved the pedagogical view that knowledge and awareness of the working of one’s own cognitive system —acquired by reflection on one’s own *learning processes*— would improve student’s learning abilities and eventually, provide them with *metacognitive skills*.

Thus, initially the notions of metacognitive knowledge and skills were focused on students’ *learning abilities*, disconnected from the ability to understand and use (scientific) knowledge in scientific research and problem-solving tasks. But in the work of later authors, the notion of metacognitive skills becomes entangled with accounts of what it means to (deeply) understand scientific knowledge as part of having acquired *expertise* in research (esp. in the philosophical work by Goddiksen and Andersen 2014, and Goddiksen 2014). This turn is crucial to our own argument, which aims at an epistemological view that recognizes the contribution of the human cognitive system and specificities of scientific fields (MacLeod 2016) to the character and form of (scientific) knowledge (Boon 2017b). In particular, the *metacognitive knowledge* must include knowledge of the epistemological nature of (scientific) knowledge, and accordingly, the metacognitive skills of an expert are the higher-order cognitive skills to use this metacognitive knowledge to understand, organize and execute (interdisciplinary) research processes. In Section 4, it will be argued that metacognitive *scaffolds* represent metacognitive *knowledge* – these scaffolds (also referred to as *matrices* and *frameworks*) can be utilized to learn and execute (interdisciplinary) scientific research, i.e., to develop metacognitive *skills* in doing research.

Our interrelated epistemological and educational view is on par with that of those authors in the educational sciences who indeed argue that the dominant *positivist* view of science hampers the development of metacognitive or higher-order cognitive skills, and who therefore promote the learning of these skills in a direct relationship with alternative epistemological views – often called *constructivist* views (e.g., Edmondson and Novak 1993; Yerrick et al. 1998; Procee 2006; Tsai 2007; Mansilla 2010; DeZure 2010; Zohar and Barzilai 2013; Abd-El-Khalick 2013; Sin 2014; and see footnote 9).

The core of our philosophical approach aiming to clarify teaching and learning higher-order cognitive skills is to focus on how researchers (and more generally, cognitive and epistemic agents) *construct knowledge* – which involves an epistemological view that we aim to express by the suggested *engineering-design* metaphor of knowledge-generation in interdisciplinary research, in contrast with the *jig-saw puzzle* and the *conflict-resolution* metaphors. The proposed epistemological view entails that ‘knowledge’ must not be understood as a literal *representation* of aspects of the world (as is implicit in the jig-saw puzzle metaphor) independent of how researchers trained within a scientific discipline typically construct knowledge,^8^ but rather as shaped by researchers in ways learned within their specific scientific discipline (i.e., as expressed by the alternative *engineering-design* metaphor). Typical ways of constructing ‘knowledge’ within a discipline is conceptually grasped by the notion ‘disciplinary perspective.’

The proposed epistemological view, therefore, provides an alternative to the widely criticized *positivist* views of science (esp. see the authors listed above) still conveyed in science education,^9^ and forms the basis for alternative views on teaching and learning higher-order cognitive skills, in particular, regarding the role of metacognitive scaffolds (frameworks) to support developing and executing these skills (also see section *Terminology*). The core of our philosophically supported educational view is that to develop expertise in conducting interdisciplinary research, students need to: (1) learn and understand *that* knowledge is *constructed*, rather than being a literal representation independent of the disciplinary context and independent of typical (disciplinary) ways in which researchers construct knowledge; (2) learn that, (at least partially) knowing *how* specific knowledge is generated or constructed, is often crucial to understanding *how to use* this knowledge in problem-solving tasks; (3) learn to recognize, or actually reconstruct in a systematic fashion, *how* specific knowledge has been constructed; (4) understand that scientific disciplines have developed different ways to generate knowledge, which is grasped by notions such as *disciplinary paradigms*, *matrices* and *perspectives* (Spelt et al. 2009); (5) learn to understand the *coherence* of epistemic norms and activities in scientific practices (Chang 2012, 2014); and, (6) learn to recognize, or actually reconstruct in a systematic fashion, the specificities of a scientific discipline (Andersen 2013, 2016).

## Philosophical view of science

### The unity of science versus disciplinary perspectives

A dominant view of science in the traditional philosophy of science is reflected in the lack of philosophical interest in *interdisciplinarity* as a subject for philosophical study. Conversely, characteristic of this dominant view is the general interest in the *unity of science* understood as coherence between the sciences (esp., between scientific theories, laws and concepts in distinct fields or disciplines), where interdisciplinary research is merely seen as a means to achieve this unity (Oppenheim and Putnam 1958; Nagel 1961; Maull 1977; Darden and Maull 1977; Grantham 2004; Schmidt 2008, 2011; Thorén and Persson 2013; Cat 2014). The *jigsaw-puzzle* metaphor of how interdisciplinarity is achieved, therefore, can also be taken as a metaphor of how the *unity of science* is viewed.

Another characteristic of dominant traditional philosophical views of science is the lack of philosophical interest in epistemological issues of generating epistemic resources for solving problems in the real world, and the role of interdisciplinary research therein.^10^ This neglect is reflected in much of academic science education, which traditionally pays little attention to the *use* of science in constructing epistemic sources (such as models and concepts) for specific epistemic tasks. Even today it is difficult to encourage bachelor students in the engineering sciences to construct scientific models for real-world target systems. They are trained in constructing mathematical models (especially for the exercises in textbooks), but hardly able to (re)construct scientific models in the sense of searching and putting together epistemic resources into a coherent (preliminary) scientific model (Boon and Knuuttila 2009; Knuuttila and Boon 2011; Boon 2019; Newstetter 2005). On this part, we suspect that students often have a confused understanding of (non-mathematical) scientific models because of a naive *representational* understanding of models in which models must be similar to aspects of the world. Such an understanding makes it very hard to construct, use and adapt scientific models for real-world problem-solving tasks. Philosophical views of science assuming that the ultimate aim of science is justified theories may have been one of the causes of this confusion. Social-constructivist views about science, such as the current consensus view on the ‘nature of science’ (NOS), which is supposed to be generally taught as a correct understanding of science, does not help in this respect (see footnote 9). It is, therefore, an important task for the current philosophy of science to come up with adequate alternative views.

Finally, with regard to the characteristics of dominant views of science that may have affected the view of science still widely ingrained in higher education, it is striking that Kuhn’s profound understanding of the contribution of *disciplinary perspectives* —which inherently and indelibly shape the scientific results of a discipline or field— has been interpreted as a problem and a threat that must be countered to restore the objectivity, rationality and unity of science. Instead, we claim, this understanding should be firmly embraced to learn more about the consequences for the epistemology of science. More specifically, Kuhn’s insights could have led to the recognition of serious cognitive and epistemological challenges of interdisciplinary research, which deserve philosophical study (Andersen 2013, 2016). However, the jigsaw-puzzle metaphor of interdisciplinarity, and with it a naïve idea of the ‘unity of science,’ remained dominant,^11^ and is obviously *incommensurable* with notions such as ‘disciplinary perspectives.’ Conversely, we defend that notions such as ‘disciplinary perspectives’ are productive to better understand the character of science and actual scientific research in scientific practices.

It appears, therefore, that philosophical views of science held within the philosophy of science determine which issues are recognized as of philosophical interest. However, as these views also affect views of science in society at large, especially in higher education and scientific research, it is important to critically examine ‘philosophical paradigms of science,’ that is, philosophical paradigms of science ingrained in the *philosophy of science* and beyond.

### Two philosophical paradigms of science: A physics versus an engineering paradigm of science

At stake are two different *philosophical* views on science, one focusing on scientific theories for the sake of science,^12^ the other focusing on scientific knowledge (in the sense of epistemic resources and results, see *Terminology*) and epistemic strategies for solving real-world problems. Boon (2017a) has argued that these two views can be analyzed in terms of two distinct *philosophical paradigms of science*.

The core of a Kuhnian notion of paradigms *in* science —as we interpret it in regard of the epistemological issues raised in this article— is that a scientific practice (or discipline) is embedded in a paradigm that enables and guides it, rather than being guided by strict methodological rules alone.^13^ Conversely, the paradigm is ingrained in the sense that the practice (or discipline) maintains and reinforces it. The paradigm frames what counts as relevant scientific problems and adequate solutions, as well as how these problems are phrased, and also how the discipline deals with it. Although a paradigm cannot be proven or disproven in a straightforward manner, it can be articulated, analysed and disputed, for which the *disciplinary matrix* introduced by Kuhn (1970) is suggested as an analytic framework.^14^ In this ‘disciplinary matrix’ explication, a paradigm consists of a loose, non-rigid set of interlocking elements that mutually support and reinforce each other.

The same can be said of *philosophical* paradigms *of* science, being views of science that guide and enable *philosophical studies* of science – i.e., the philosophical paradigm frames what counts as relevant philosophical problems and adequate solutions, how these problems are phrased, and how the philosophy of science deals with it (Boon 2017a). Accordingly, similar to, and based on Kuhn’s disciplinary matrix by which philosophers can analyse the philosophical fabric of *scientific* disciplines, a matrix (or framework) has been developed for analysing *views of science in the philosophy of science*. Expanding on Kuhn’s disciplinary matrix that consists of four elements, thirteen elements that constitute a matrix for analyzing philosophical views of science have been proposed (listed in the left column of Table 1). Subsequently, the resulting matrix has been used to articulate two contrasting philosophical views of science, called a *physics paradigm* and an *engineering paradigm* of science, of which a sketchy summary is presented in the right column of Table 1 (for a more elaborate version, reference is made to Boon 2017a).

**Table 1:** Matrix to articulate and analyze *philosophical views of science* (constituted by elements listed in the left column), and a summary of a physics paradigm of science versus an engineering paradigm of science (presented in the right column; based on Boon 2017a, with minor changes and additions)

**Elements constituting the matrix**	**Physics paradigm versus Engineering paradigm**
I. Epistemic aim(s) of scientific research => science aims at … versus scientific research aims at …:	e.g., true or adequate theories, which describe or represent ‘what the world is like’ (in realism) or which ‘save the phenomena’ (in anti-realism), versus functional *epistemic tools* for epistemic tasks.
II. Epistemic values and (pragmatic) criteria for the acceptance of knowledge (similar to Kuhn’s epistemic values) => science results must meet criteria such as … versus scientific research must meet criteria such as …:	e.g., truth or empirical adequacy; universality and coherency between theories; simplicity; explanatory & predictive power; (internal) logical consistency; derivability of knowledge at higher levels from knowledge at lower levels; and, testability or falsifiability, versus empirical adequacy; reliability and relevance in view of epistemic purposes (for practical uses); simplicity in the sense of manageability & tractability; intelligibility; balance between generality & specificity in view of epistemic aims; explanatory & predictive reliability; logical consistency; coherence with accepted knowledge relevant to epistemic uses; integration of (heterogeneous bits of) knowledge from different fields and levels; validation in view of epistemic uses and functions.
III. Basic and ‘regulative’ principles (i.e., basic assumptions and rules guiding scientific research, Boon 2015):	e.g., unity of science; reductive explanation; generalization (inductive inference); and, invariance, versus disunity (e.g., Cartwright’s 1999 *Dappled World*); generalization based on ceteris paribus or ‘same conditions-same effects’; invariance; and, *construction* (rather than logical or mathematical derivation) of explanatory models.
IV. Theoretical principles of a discipline (similar to Kuhn’s symbolic generalizations), i.e., what, according to philosophers, counts as such:	e.g., axiomatic theories; fundamental principles; and, laws of nature, versus axiomatic theories; fundamental principles; laws as tools in model-building; and, scientific concepts and models as tools to (experimentally) investigate phenomena and technological instruments (producing these phenomena).
V. Metaphysical pre-suppositions (similar to Kuhn’s metaphysical presuppositions):	e.g., the world has a hierarchical structure and is well-ordered; and, *physicalism*, versus the world has a complex, non-hierarchical structure; and, phenomena do not exist independently.
VI. Ontology (i.e., how the subject-matter of research is conceptualized):	e.g., the physical world consists of objects, their properties and their causal workings; and, *ontological reductionism*. This agrees with metaphysical presuppositions, i.e., that higher-level objects, their properties and their causal behaviour supervene on lower-level physical objects and properties, versus physical phenomena are conceptualized in terms of their (e.g., biological or technological) functions; typical engineering concepts are used in these functional ‘descriptions’; and, *operational definitions* of (unobservable) phenomena inherently encompass aspects of instrumental and experimental set-ups typical of the discipline in which they are investigated (Boon 2012).
VII. Subject-matter (i.e., types of ‘things’ studied in scientific research, which is close to ‘ontology’ but more concrete and discipline-specific) => science aims at (explaining) … versus scientific research aims at (explaining, modeling, generating) …:	e.g., physical or biological phenomena (‘in nature’), versus naturally or technologically produced or producible phenomena and instruments.
VIII. Epistemology => research in science is (normatively) guided by … aiming at …., versus scientific research is (normatively) guided by …. aiming at …:	e.g., *theoretical and explanatory reductionism*; *why-necessary* explanations are better than *how-possible* explanations; and, scientific results such as models must be ‘truthful’ *representations*, versus, constructing knowledge (e.g., concepts, models); *how* and *how possible* explanatory laws and models, which are ‘epistemic tools’ that must allow for epistemic uses in view of epistemic tasks (e.g., in real-world problem-solving).
IX. Methodology => scientific research adopts as proper methodology … versus …:	e.g., *methodological reduction* motivated by metaphysical, ontological and epistemological presuppositions, versus methodological reductionism as a pragmatic strategy next to other strategies to investigate subjects of interest.
X. Exemplars of science (rather than exemplars of theories as in Kuhn’s matrix):	e.g., theoretical physics and physical chemistry, versus synthetic biology; and, interdisciplinary fields such as traditional engineering sciences, nanoscience and technology, and biomedical sciences.
XI. Role attributed to experiments and technological instruments:	e.g., to discover new (physical) phenomena and to test hypotheses, versus to discover and create physical phenomena (that eventually may be of functional interest); and, the technological production of functional phenomena, where also the technological instruments are object of research and development.
XII. Results of scientific research => science *is* … versus scientific research aims at epistemic results such as …:	e.g., theories, laws, and phenomena, versus data-sets; technologically produced phenomena; phenomenological laws; scientific concepts; technological instruments and experimental model-systems; and, scientific models of both phenomena, technological instruments, and (mechanistic) workings of experimental set-ups and technological instruments.
XIII. Justification (i.e., how and why results are justified, accepted, and tested) => science aims to … versus scientific research aims at …:	e.g., test (confirm or falsify) a hypothesis, basically through hypothetical-deductive-like methods, versus validation of epistemic results in view of intended epistemic uses (also regarding pragmatic criteria such as intelligibility), where much of the justification is already ‘in place’ based on discipline-specific ways of constructing ‘knowledge,’ (Boumans 1999).

Crucially, the elements of the matrix constituting and representing a philosophical view of science are intertwined and reinforce each other, to the effect that alternative (philosophical) views on specific aspects of science do not easily get a foothold in the philosophy of science. This is why it is considered a paradigm – it is a comprehensive as well as normative and more or less implicit background view within which philosophical ideas about science are articulated, understood and evaluated.

Although the suggested *physics paradigm of science* might be dominant as a view of science (implicitly) adopted by philosophers of science but also by scientific researchers (which is especially obvious in roles where they have to *articulate* their views of science, such as when teaching or when being interviewed by philosophers), reasons can be given that an *engineering paradigm of science* is more adequate for characterizing actual scientific research practices. In particular, scientific practices aiming at knowledge that is relevant, reliable, and useful for solving real-world problems, are better understood within an engineering paradigm of science.

Contrariwise, most of the traditional philosophy of science considers these kinds of research practices as ‘applied sciences,’ in the sense that these ‘problem-solving’ practices ‘simply’ apply scientific knowledge generated in fundamental or basic sciences (Boon 2006, 2011). This belief is intertwined with, and strongly supported by the suggested physics paradigm of science in regard of elements listed in Table 1 such as the aim of science, ontology, metaphysical presuppositions, methodology, (epistemic) results of scientific research, and the role of technological instruments, which are interpreted differently in an engineering paradigm of science.

### The physics paradigm as a cause of epistemological difficulties of interdisciplinary research?

Does the physics paradigm of science cause philosophical misunderstanding of epistemological difficulties of interdisciplinary research? And if so, is the engineering paradigm doing better?

A reason for this hypothesis is that on the physics paradigm, science aims at theories and (reductive) unity of science, which implies that the aim and results of *interdisciplinary* research must be phrased in terms of *integration* of theories, as can be observed indeed in most authors who study interdisciplinarity discussed in this article. Above, this flawed understanding of interdisciplinary *integration* has been criticized as the jigsaw-puzzle metaphor of interdisciplinarity. Some authors have already criticized the idea that unity of science must be achieved by means of reductive relationships between theories.^15^ We take Maull (1977) and Darden and Maull (1977) as in fact pointing at the possibility to draw relationships between disciplines by the exchange of theories and concepts *without* integration.^16^ Similarly, Thorén and Persson (2013) have introduced the notion of ‘problem-feeding,’ which is also a way in which collaboration between disciplines can take place without integration proper. Our point is that interdisciplinary research as a means to achieve *unity of science* through *integration* of the theoretical content of disciplines fits with a physics paradigm, whereas interdisciplinary research in the sense of collaborations between scientific disciplines aimed at ‘epistemic tools,’ methodologies, and (technological) instruments that can eventually serve as epistemic tools for solving problems outside the disciplines, does not fit well into a physics paradigm, but rather into the engineering paradigm.

A more fundamental issue is to see how *disciplinary perspectives* cause epistemological and cognitive difficulties in interdisciplinary research. Several authors stress the importance of *disciplinary perspectives* in interdisciplinary collaborations, either in terms of the rigidity to conceptual or theoretical change (e.g., Thorén and Persson 2013), or by indicating the importance of recognizing that different perspectives are possible on the same problem. However, eventually most authors assume that this can be dealt with by *communication* and finding ‘*common ground*’ among disciplinary perspectives on a problem (e.g., DeZure 2010; Ivanitskaya et al. 2002; Aram 2004; Repko et al. 2007; Spelt et al. 2009; Haynes and Brown-Leonard 2010; Liu et al. 2011; Lattuca 2001, 2002; Lattuca et al. 2017).

Our concern is that the pursuit of ‘common ground’ by unassisted communication in interdisciplinary research is very difficult or remains at a superficial level – a concern that is also supported by empirical research in the educational sciences discussed above. Conversely, interdisciplinary collaboration may become more effective by better understanding how *disciplinary perspectives* work in *disciplinary* scientific research.

First, the contribution of disciplinary perspectives cannot easily be appreciated within a physics paradigm of science, in which it is assumed that science aims at theories that objectively *represent* aspects of the world, that is, as a two-placed relationship between knowledge and world – an assumption that smoothly agrees with the jigsaw-puzzle metaphor of interdisciplinary research. In our explanation of the role of disciplinary perspectives in generating knowledge within a discipline, we take *scientific models* as an example. A widely discussed issue in the philosophy of science is how scientific models *represent* their target-system. A favoured account, also in science education and communication, is that it consists of a similarity relationship (e.g., Giere 1999, 2004), which however appears problematic. In order to avoid the problematic aspects of similarity, both Giere (2010) and Suárez (2003, 2010) develop an account which attributes a key-role to the *competent and informed agent*. However, their accounts are still not very informative as to the epistemic functioning of models (Knuuttila and Boon 2011).^17^ Also ‘context-dependence’ is often mentioned to indicate that an objective (two-place) representational relation is problematic, but with a few exceptions,^18^ hardly any of these studies explains how the ‘context’ or the disciplinary perspective of the epistemic agent contributes to the epistemic character of the representation. We aim to open this ‘black-box,’ as we believe that this is a way in which philosophers can contribute to difficulties of interdisciplinary research.

Boon and Knuuttila (2009) and Knuuttila and Boon (2011) have turned focus to how models are *constructed*, and argue that several heterogeneous elements are built-in the model, which partly but indelibly determines ‘what the model looks like.’ Constructing a scientific model typically occurs within a specific discipline and focuses on a problem within or outside the disciplines. Researchers usually pick a specific aspect of the problem (the phenomenon of interest) for which the model is build. This choice is guided by the disciplinary perspective. Next, the way in which the model is constructed is guided, enabled and constrained by what the discipline has to offer, which concerns aspects such as: the experimental set-up by which the phenomenon can be studied; the instruments which determine what can actually be measured; the available theoretical and empirical knowledge about the phenomenon; and, the kind of simplifications usually made in the discipline (e.g., due to recommended ‘methodological reductions’), both in the experimental investigation and in the construction of the model.^19^ This brief sketch illustrates that scientific models constructed in this manner cannot be understood as a straightforward representations in the sense of a two-placed relationship between model and target-system, nor can they be understood as mathematically derived from abstract theories (although parts of the model may be derived in that way). Boon/Knuuttila (2009, 2011) have argued that scientific models usually are representations in the sense of being *epistemic tools* that allow for thinking and reasoning about the target system, rather than being representations in the sense of firstly being *similar* to the target system. Whereas the actual epistemic uses of scientific models are unintelligible when assuming that the model is *similar* to its target, epistemic uses of scientific models by scientific researchers are better understood when taking into account how the mentioned aspects have shaped the model (see footnote 19). As has been argued in Boon (2017a), the interpretation of *scientific models as representation* complies with the physics paradigm of science, while the notion of *scientific models as epistemic tool* is virtually unintelligible within the physic paradigm. Conversely, the notion of knowledge as epistemic tool is a core feature of the engineering paradigm of science.

Regarding the question “Does the physics paradigm of science cause philosophical misunderstanding of epistemological difficulties of interdisciplinary research, and if so, is the engineering paradigm doing better?” it can now be answered that, firstly, the physics paradigm considers scientific knowledge such as models as objective representations independent of how these representations are shaped by the specific scientific discipline. This makes it very hard for experts trained in D_B_ to understand epistemic resources produced by experts trained in D_A_. Conversely, the engineering paradigm takes into account that aspects of a scientific practice fundamentally shape scientific knowledge. For instance, the suggested method for (re)constructing scientific models (Boon 2019; see footnote 19) enables to analyze the aspects that a discipline typically builds-in the model. Within a physics paradigm of science, this contribution of aspects specific to the discipline in shaping epistemic results leads to concerns about the objectivity of knowledge. Yet, this is much lesser of a concern within the engineering paradigm, because an important criterion for accepting epistemic results is rather that the knowledge must be constructed such that it can properly function as an epistemic tool in performing epistemic tasks, for instance with respect to solving real-world problems.

## Metacognitive scaffolds

### Disciplinary matrices and disciplinary perspectives as metacognitive scaffolds

On the proposed Kuhnian approach, a *disciplinary perspective* of experts in a specific discipline D_A_ can be made explicit by means of the elements that constitute the *disciplinary matrix*, that is, in terms of a more or less coherent set of knowledge, beliefs, values and methods that has become ‘second nature’ in the sense that experts are hardly aware of how the specificities of their disciplinary contribute to the ways in which they do their research and generate *epistemic results*. Being trained in discipline D_A_ has instilled in the researcher a disciplinary perspective specific to D_A_, which basically *enables* but also *constrains* how she does her research. When facing, say, a ‘real-world’ problem, the disciplinary perspective makes her *observe* phenomena P_A_ typically dealt with in D_A_. Hence, researchers working in D_A_ observe some aspects P_A_ of the problem, but maybe not aspects P_B_ that would be typically observed by experts trained in discipline D_B_. Next, the disciplinary perspective makes researchers phrase *research questions* typical of D_A_, and construct *explanations*, *models* and *hypotheses* about P_A_ by means of *epistemic resources* and *epistemic strategies* typical of D_A_. Also, researchers investigate P_A_ by means of *measurement procedures* typically used in D_A_, and design *experimental set-ups* and *technological instruments* in ways typical of D_A_ as well. Finally, *epistemic results* about P_A_ are *tested* by *procedures* also typical of D_A_.

This brief sketch of what a *disciplinary perspective* of a discipline D_A_ consists of, results in a set of specific elements that constitute a disciplinary matrix, such as: phenomena; research questions; epistemic resources, e.g., fundamental principles, theoretical and empirical knowledge; epistemic strategies; methods and methodologies, e.g., statistical analysis; experimental and technological setups; and, measurement instruments. To this rather preliminary and intentionally ‘not rigid’ pragmatic list of elements, some elements pointing at deeper philosophical issues listed in Table 1 can be added, for instance when aiming to find out about differences caused by philosophical presuppositions such as may be at stake in collaborations between, for instance, the humanities, social sciences, natural sciences, and the engineering sciences. In short, we suggest that the *disciplinary perspective* of a specific discipline D_A_ can be analyzed in terms of such a set of cohering elements, called a *disciplinary matrix*.

The disciplinary matrix can be considered as a *metacognitive scaffold*, or *framework* that enables researchers to characterize their own disciplinary perspective in terms of a limited set of concrete aspects typical of their discipline. These aspects can, for example, be used to clarify approaches of the discipline. In interdisciplinary collaborations, this approach can be used to communicate with experts from other disciplines, for instance, in order to find similarities and differences in presuppositions and approaches of D_A_ as compared to D_B_. In short, the disciplinary matrix to articulate and investigate disciplinary perspectives functions as a metacognitive scaffold that facilitates interdisciplinary communication on the characteristics of each discipline involved in an interdisciplinary research project. It is a scaffold that helps to open up disciplinary silo’s.^20^

Using the term *disciplinary perspectives* of D_A_ —but even more so, the term *metacognitive scaffolds*— may suggest a rather ‘immaterial,’ cognitivist take on the contribution of the specificities of a discipline D_A_ in shaping the results and the form of the results. Yet, crucially, also technological instruments used in D_A_ are an inherent part of the disciplinary perspective, not as ‘windows on the world’, but, for example, in already shaping and even generating phenomena that would not exist without these instruments (Giere 2006; Van Fraassen 2008; Boon 2012, 2017c). By referring to disciplinary perspectives, we wish to stress the contribution of the disciplinary perspective to the specificities of the research outcomes. It is to stress that these aspects of the disciplinary perspective (indicated by the elements of the disciplinary matrix) partially determine what the ‘knowledge’ produced by a specific discipline D_A_ ‘looks-like’ – to stress that this knowledge is not a representation of the studied phenomenon, independent of specificities of the scientific discipline that produced this knowledge.

Acknowledging the contribution of the specificities of a discipline to the epistemic (and technological) results of scientific research stresses why metacognitive scaffolds (and the skills to use these scaffolds) are crucial to the researcher in interdisciplinary settings: For a researchers unfamiliar with D_A_, the epistemic resources produced by discipline D_A_ do not speak for themselves, as ‘knowledge’ is not a straightforward representation of what the world is like. Instead, to understand ‘knowledge’ of unfamiliar disciplines requires the ability to also recognize it as resulting from specific ways of thinking, experimenting, measuring and modeling within discipline D_A_. The method of using the disciplinary matrix and disciplinary perspectives as metacognitive scaffolds helps in understanding an unfamiliar discipline D_B_ in terms of the elements that guide and confine the way in which researchers in D_B_ approach their subject and construct ‘knowledge.’ It explains the ‘how’ of research in D_B_ by means of which ‘knowledge’ (the ‘what’) used and produced in D_B_ is more easily understood.

### Frameworks: (disciplinary) matrices and metacognitive scaffolds as epistemic tools

A core idea of the engineering paradigm of science is to interpret epistemic entities such as axiomatic systems, principles, theories, laws, descriptions of (‘unobservable’) phenomena, scientific models and concepts as *epistemic tools* that can serve in epistemic activities aimed at specific (epistemic) purposes. It is in view of their epistemic *functioning* that constructed epistemic tools must meet specific epistemic and practical criteria.

As already appears above, additional to interpreting ‘knowledge’ as epistemic tool, we propose to also interpret frameworks such as (*disciplinary) matrices* and *metacognitive scaffolds* as epistemic tools that are constructed and designed (e.g., by philosophers but also researchers) to support students in their learning (to understand science) as well as researchers in performing epistemic tasks (see footnotes 19 and 20 for additional examples of metacognitive scaffolds). This suggestion complies with the engineering paradigm but not very well with the physics paradigm. In the latter, frameworks such as (disciplinary) matrices and metacognitive scaffolds are assessed as to how well (truth-full) they *represent* their target, whereas the engineering paradigm stresses that they must be assessed as to how well they serve a specific epistemic function (e.g., of the ‘system of practice,’ Chang 2012), that is, how well they serve as (epistemic) tools in performing epistemic tasks. As a consequence, in an engineering paradigm of science, disciplinary matrices, disciplinary perspectives and even philosophical paradigms are assessed for how well they function in a specific (scientific, practical, problem-solving) context.

### Interdisciplinary research

In this article, we have aimed to make plausible that epistemological difficulties of interdisciplinary research have to do with dominant philosophical beliefs about science. The core of our argument is that scientific knowledge is usually presented as if it results from a representational relationship between knowledge and world, ignoring the role of disciplinary perspectives. Such an approach may be relatively unproblematic as long as we stay within the confines of a discipline and expect that every newcomer ultimately adapts to the specificities of the discipline. Surely, most researchers have at least some understanding of what it means to have a disciplinary perspective, but working within the confines of their well-established scientific discipline they hardly need to take into account that scientific results are shaped by the specificities of their discipline. Yet, this situation causes wicked problems as soon as interdisciplinary cooperation is requested.

The suggested solutions is to adopt an epistemological view in which scientific knowledge (such as models) is understood as also shaped by the specificities of the discipline. More effectively dealing with the specificities of scientific disciplines in interdisciplinary collaborations may be require meta-cognitive scaffolds (and the ability to use them) that enable analyzing how exactly a discipline generates and applies knowledge. Three examples of these scaffolds have been briefly sketched: the disciplinary perspective of a specific discipline can be analyzed and articulated by means of a (disciplinary) matrix; the way in which models are constructed can be analyzed and articulated by the so-called B&K method  (Boon 2019); and the way in which scientific concepts are embedded in a wider context can be analyzed by means of concept-mapping (see footnote 20).

Therefore, not *integration* of theories and disciplinary perspectives is the first task for interdisciplinary collaboration, but clarification of the specificities of the disciplines and of the way in which in a discipline ‘knowledge’ comes about.

## Footnotes

1 Conversely, Kline (1995) stresses that the complexity of a problem is often only recognized when it is studied from multiple perspectives.

2 Some authors, such as Schmidt (2008, 2011), use interdisciplinarity and transdisciplinarity interchangeably.

3 Below, we will suggest that the aims of teaching interdisciplinarity expressed in engineering and sustainability education correspond better to the definition of ‘transdisciplinarity.’

4 Schmidt (2008, 2011) has proposed a typology to distinguish between the different types of *problems* that are addressed in interdisciplinary (and transdisciplinary) research. He calls this object-oriented, theory-oriented and method-oriented interdisciplinarity, versus problem-solving oriented interdisciplinarity. His paradigmatic example of the latter is sustainability science, whereas ‘instrumental’ interdisciplinary approaches in the engineering sciences (which are the focus of our article) fall into the category ‘method-oriented’ interdisciplinarity. Attempts to address the problem of unity and the interconnections within the sciences are covered by object-oriented interdisciplinarity (if someone leans to a realist position on science with respect to ontology) or theory-oriented interdisciplinarity (if one tends towards an anti-realist position focused on epistemology). It is not our intention to discuss this at length, but for our purpose, on the one hand, Schmidt’s methodology-oriented interdisciplinarity is too limited to characterize the engineering sciences, while on the other hand his problem-oriented interdisciplinarity is too much oriented at societal issues, and thus runs the risk of neglecting the specific cognitive and epistemological difficulties of interdisciplinary research aimed at complex ‘real-world’ problems.

5 In educational settings the hypothetical-deductive method is often referred to as the *empirical cycle*.

6 For instance, in national and international research programs, such the European Horizon2020 program on grand societal challenges: https://ec.europa.eu/programmes/horizon2020/en/h2020-section/societal-challenges

7 The problem that rules for the uses of theories in real-world problems are not given by the theory was already stressed by Cartwright (1983).

8 Nor must the generation of knowledge in interdisciplinary research be understood as the mere product of social deliberation, more or less independent of ‘what the world is like,’ as suggested in strong *social-constructivism*. It should be noted that by using the *conflict-resolution metaphor,* we do not intend to attribute a strong social-constructivist position to scholar such as Klein, Repko and Szostak, as these authors maintain a much more moderate position. Furthermore, our focus is on the natural and engineering science, whereas many authors studying interdisciplinarity also aim to cover the humanities and social sciences, where the conflict-metaphor is probably more appropriate.

9 The alternative epistemological view proposed here, also aims to improve on *constructivist* views explicitly promoted in the so-called *nature of science* literature. Although the widely adopted view on the *nature of science* in science education literature stresses the social character of science as well as the role of human values in science, it still reinforces what educational scientists often call a *positivist* view of science regarding the character of knowledge. McComas et al. (1998) present a comprehensive list that summarizes the established view on the *nature of science* (NOS) that must be taught in science education. In recent literature, this so-called consensus view on the NOS has remained mostly unchanged.

10 Notable exceptions is the early work of Cartwright, Nersessian, and Dupré, and see more recent work by Mattila 2005; Mitchell 2009; Frodeman 2010; Green 2013, Grüne-Yanoff 2011, 2014; and Holbrook 2013. Also see the work by Andersen, Goddiksen, Nersessian, MacLeod, Schmidt, and Thorén referred to in this article.

11 Nonetheless, the *unity of science*, has been disputed already at an early stage by dissidents such as Dupré (1983), and also see Mitchell (2009).

12 On this view, science *is* scientific theories.

13 See Andersen (2013, 2016) for a comprehensive explanation of Kuhn’s ideas related to disciplines and interdisciplinarity.

14 Our approach to the philosophy of science agrees in many respects with Chang (2012, 2014) who argues that “serious study of science must be concerned with what it is that we actually *do* in scientific work. … Scientific work consists in actions, carried out by agents. An agent carries out intentions. A scientist is not a passive receiver of facts or an algorithmic processor of propositions. … [Therefore,] in serious study of science, we need to consider human capabilities (capacities and skills) in performing epistemic activities” (Chang 2014, 70). Chang (2012) proposes the notions *epistemic activities* and *systems of practice* – i.e., a system of practice is formed by a coherent set of epistemic activities performed in view of aiming to achieve certain purposes. Additionally, it is the overall purposes of a system of practice that define what it means for the system to be *functionally coherent*. Yet, Chang (2012) argues that the notion ‘system of practice’ is better suit for the analysis of practices than Kuhn’s notion ‘disciplinary matrix’ because, according to Chang, it is not clear how the elements of Kuhn’s matrix hang together. In our view, Chang adds important insights, especially by emphasizing the role of epistemic activities in scientific practices and the overall purpose of a system of practice together with the idea of ‘functional coherence’ between epistemic activities in forming a system of practice, but we suggest to consider Kuhn’s notion of ‘disciplinary matrix’ and Chang’s notion of ‘system of practice’ as complementary to a better understanding scientific practices. Additionally, contrary to Chang, we claim that there exists coherence between the elements of the matrix in the sense that these elements mutually support and reinforce each other, which is why it functions as a paradigm.

15 Admittedly, assuming reductive relationships between theories, as in traditional unity of science views (Oppenheim and Putnam 1958; Nagel 1961), makes intelligible how *integration* in science takes place.

16 Maull (1977) and Darden and Maull (1977) introduced the notion of ‘field’ to enable talk about (non-hierarchical and non-reductive) interrelationships that historically develop between fields. They did not introduce ‘field’ as an alternative to ‘discipline.’ In this article, we use ‘fields’ and ‘disciplines’ interchangeably.

17 Both Giere and Suárez defend to have intentionally developed a deflationary notion of representation that only minimally characterizes the representational relationship between model and real-world target system.

18 Giere’s (2006) and Van Fraassen’s (2008) account of representation are important contributions to developing this understanding, but their accounts are beyond the current scope.

19 A more systematic explanation of this so-called B&K method for the (re)construction of scientific models is presented in Boon (2019). The B&K method helps researchers to understand scientific models in an unfamiliar discipline D_A_ in terms of the a list of elements that guide, enable and confine the way in which researchers in D_A_ construct models. Therefore, this list of elements indicates the kinds of aspects that determine the disciplinary matrix, and the suggested method of (re)construction scientific models can be interpreted as a metacognitive scaffold to assist in interdisciplinary communication – in this case, to learn how discipline D_A_ typically constructs scientific models.

20 Several authors in the educational sciences who explicitly reject a *positivist* epistemology in science education, have proposed concept-mapping as a way to teach science in a more *constructivist* fashion – in this case, as a way to better understand scientific concepts as compared to traditional rote learning (e.g., Novak 1990; Weideman and Kritzinger 2003; Addae et al. 2012; Thomas et al. 2016). Concept-mapping definitely fits with the engineering paradigm. We also recognize the potential of this approach for learning scientific research. However, concept-mapping is usually introduced as a rather ‘empty’ framework, which students find hard to use. Therefore, we suggest for this approach to become an effective *metacognitive scaffold*, some more guidance is needed in how to construct a concept-map, for instance similar to the introduction of concrete elements in a matrix (left column of Table 1), or the concrete elements in the method to construct scientific models.
